# Response Surface Methodology (RSM) Optimization of the Physicochemical Quality Attributes of Ultraviolet (UV-C)-Treated Barhi Dates

**DOI:** 10.3390/plants11172322

**Published:** 2022-09-05

**Authors:** Mahmoud Younis, Isam A. Mohamed Ahmed, Khaled A. Ahmed, Hany M. Yehia, Diaeldin O. Abdelkarim, Assem I. Zein El-Abedein, Abdulla Alhamdan

**Affiliations:** 1 Chair of Dates Industry & Technology, King Saud University, Riyadh 11451, Saudi Arabia; 2 Agricultural Research Centre, Agricultural Engineering Research Institute (AEnRI), Giza 12619, Egypt; 3 Department of Food Science and Nutrition, College of Food and Agricultural Sciences, King Saud University, Riyadh 11451, Saudi Arabia; 4 Department of Food Science and Nutrition, College of Home Economics, Helwan University, Helwan 11611, Egypt; 5 Department of Agricultural Engineering, College of Food and Agricultural Sciences, King Saud University, Riyadh 11451, Saudi Arabia

**Keywords:** Ultraviolet (UV-C) post-harvest treatment, Khalal maturity stage, Barhi dates, UV-C dose, response surface methodology (RSM)

## Abstract

Barhi date fruit is one of the most important fruits that has high consumer preference and market value at the Khalal maturity stage. However, this stage is very short and the fruit is vulnerable to decay and the ripening process under improper handling and storage conditions. Thus, the purpose of this study was to evaluate the feasibility of utilizing ultraviolet (UV-C) as a method to preserve the qualitative features of Barhi dates under various storage circumstances. The core of this study was defining the best conditions for UV-C treatment of Barhi dates, which was accomplished using a response surface methodology (RSM) model with a central composite, rotating four-factors-mixed-levels design (CCRD). The impacts of independent variables [UV-C exposure time (1, 2, 3, 4 min), UV-C dose (1, 3, 5, 7 kJ/m^2^), storage time (1, 6, 11, 16, 21 days) and storage temperature (1, 5, 15, 25 °C)] on the moisture content (MC), total soluble solids (TSS), total color changes (E), firmness, total phenolic content (TPC), total viable count (TVC), DPPH antiradical activity, fructose and glucose were investigated. The results revealed that the optimum UV-C treatment and storage settings for keeping the quality features of the dates were the UV-C exposure period and dosage of 1 min and 2.07 kJ/m^2^, and the storage time and temperature of 18 days and 12.36 °C, respectively. At the optimum conditions, the values of 59.66% moisture content, 38.24% TSS, 60.24 N firmness value, 48.83 ΔE, 0.07 log CFU/g TVC, 5.29 mg GAE/g TPC, 56.32% DPPH antiradical activity, 6.87 g/100 g fructose and 14.02 g/100 g glucose were comparable predicted values demonstrating the suitability of the used RSM models. Overall, the perfect UV-C treatment and storage circumstances for extending the storability and shelf life and maintaining the quality features of Barhi dates were identified in this study.

## 1. Introduction

The date palm (*Phoenix dactylifera* L.) is one of the oldest and most valuable multipurpose trees that is cultivated in the arid and semi-arid regions in North Africa and western Asia. However, due to its adaptation to harsh environmental conditions and its various beneficial food, feed and pharmaceutical applications, its cultivation has expanded to other areas of the world [1,2]. Date palm fruits are the main commercially, nutritionally and pharmaceutically important products of the date palm [2,3,4]. However, other by-products of this vital crop are also of high commercial and nutritional significance [1,5], therefore, date palm is known as a multiuse and valued crop. Date fruits are considered an unexpansive source of various nutrients, including vitamins, essential minerals, protein, energy-dense carbohydrates, dietary fibers, and numerous bioactive compounds and secondary metabolites such as phenolic acids, flavonoids, tannins, tocopherols, procyanidins, anthocyanins, anthocyanidins, carotenoids, phytoestrogens and sterols. and they exhibit various health-improving properties [2,3,4]. These nutritional and health-promoting attributes of date fruits depend on various factors, such as the variety, fruit skin color, maturity stage, seasons, agronomical practices, harvesting and post-harvest processing, among other reasons [3]. Date fruits have five ripening stages known as: Hababouk (small green fruits, lasts for 1–5 weeks, 80–90% moisture), Kimri (immature green fruits, lasts for 14–19 weeks, 75–85% moisture), Khalal (mature firm fruits, lasts for 6 weeks, 40–60% moisture), Rutab (mature soft fruits, lasts for 2–4 weeks, 35% moisture) and Tamar (dry ripe fruits, 20–25% moisture) [2].

The Barhi date is one of the fewer date palm varieties that have higher commercial value and consumer preference at the Khalal maturity stage, due to its bright color appearance, sweet taste and crispness [6,7]. Barhi dates require special and controlled pre- and post-harvest processes because the fruits at the Khalal stage are very vulnerable to degradation, decay and rapid conversion into the Rutab stage and, consequently, the commercial value and consumer liking of Barhi dates is greatly diminished [8,9,10]. To date, pre- and post-harvest processing approaches, such as functional and edible coatings [7,11,12], modified and controlled atmospheric packaging [13,14,15], freezing and low temperature storage [8,9], infrared treatment [16] and the application of chemical preservatives [10,17,18], have been suggested as appropriate techniques for prolonging the shelf life of Barhi dates. One of the significant study topics is the utilization of innovative technologies to improve the quality features and extend the shelf life of Barhi dates [16].

Ultraviolet (UV) treatment is one of the most novel post-harvest processing technologies that has been applied to improve the physicochemical, nutritional and healthy quality attributes and to make fruits and vegetables last longer [19,20]. The effectiveness of UV post-harvest treatment for preserving fruits and vegetables is determined by several parameters, including the UV type and imposed UV dosage, UV exposure time, and the types and conditions of the treated samples [19,20]. The application of UV as a post-harvest treatment method has expanded in recent years due to its simplicity, safety, broad microbial control, energy efficiency, cost-effectiveness and phytochemical improvement potential, relatively small changes in the physicochemical properties, non-ionization and water requirements, no generation of byproducts and wastes and the ability to combine with other processing methods [20]. However, this treatment has some disadvantages, such as low penetration power, little residual effect, absorption by packaging materials, difficult adaptation to commercial and continuous processing methods and consumer concerns [20]. To date, different UV types (UV-A, UV-B, UV-C, and UV-V) have been used as post-harvest treatment methods for vegetables and fruits and, of them, UV-C is, by far, the most effective type in preserving fruits and vegetables [19]. Furthermore, UV-C is a post-harvest processing technology with great commercialization potential, due to its flexibility, simplicity and efficacy at low doses and, as a result, it has been widely employed as a post-harvest treatment for diverse fruits and vegetables [19,20,21]. Numerous previous reports have indicated that UV-C post-harvest treatments enhance the phytochemical, physical and nutritional quality attributes of tomatoes, fresh-cut paprika, spinach, red peppers, garlic, carrots, broccoli, blueberries, fresh-cut watermelon, pomegranates, grapes, strawberries and sweet cherries, and extend the shelf life of these fruits and vegetables [19,20,21]. In spite of the high potential applications of UV-C, its application for conserving the quality attributes and freshness of Barhi dates is limited. In order to retain the nutritional, physicochemical and microbiological quality aspects of Barhi dates, this investigation employed the response surface methodology (RSM) model to optimize the UV-C handling and storage characteristics.

## 2. Results and Discussion

### 2.1. Fitting the RSM Models

The physiochemical quality characteristics of Barhi dates (moisture content, total soluble solids, total color change, firmness, total phenolic content, DPPH antiradical activity, total viable count, glucose and fructose) were preserved during storage at various temperatures and times using a central composite rotatable design (CCRD), which was used to optimize the ultraviolet (UV-C) post-harvest treatment. The coefficient of the second-order polynomial equation was calculated using the experimental data of the total soluble solids, moisture content, firmness, total color change, total viable count, total phenolic content, DPPH antiradical activity, glucose and fructose (Equation (1)) to assess the model significance. The results of the analysis of variance (ANOVA) revealed that the models used were significant for all the quality criteria evaluated (Table 1). The adequacy, precision and repeatability of the models for all responses were evaluated by validating the coefficient of determination indicators (R^2^, modified-R^2^ and the coefficient of variation), adequacy precision and lack-of-fit. The *p*-values for the lack-of-fit of all responses ranged from 0.106 (glucose) to 6.720 (firmness), showing a non-significant (*p* > 0.05) lack-of-fit of the applied RSM models. The model’s *p*-value was also significant (*p* < 0.05) for glucose and fructose, highly significant (*p* < 0.01) for firmness, total color change (ΔE), DPPH antiradical activity and total viable count (TVC), and extremely significant (*p* < 0.001) for the moisture content (MC), total soluble solids (TSS) and total phenolic content (TPC). Moreover, the CV of all assessed attributes ranged between 0.017 (TVC) and 5.025 (ΔE), and the adequacy precision ranged from 5.386 (ΔE) to 216.80 (MC). Furthermore, the R^2^ was in the range of 0.900 (glucose) to 0.987 (TSS), and the adjusted R^2^ was between 0.811 (TPC) and 0.927 (TSS). An adequate, precise and reproducible model is characterized by a non-significant lack-of-fit, high R^2^, comparable adjusted R^2^ to fitted R^2^, high adequacy precision (>4.0) and low CV (<10%) [22,23]. Therefore, the results of this study demonstrate that the applied RSM models were adequate, precise and reproducible, and are suitable for the optimization of the ultraviolet post-harvest treatment of Barhi dates for preserving the quality features of these perishable fruits during the handling and storage.

### 2.2. Effect of Storage Conditions and UV-C Treatment on the Total Soluble Solids (TSS) and Moisture Content

The significant (*p* < 0.05) terms in the model equation obtained for the TSS were the linear terms of the storage time and storage temperature, the interaction terms of the storage time and UV-C dose, the storage temperature and time, and the quadratic term of storage time in positive and negative manners, suggesting increasing and decreasing effects on the TSS (Table 1). The other factors did not show any significant effects on the TSS of Barhi dates and the polynomial equation for the TSS after removing non-significant terms is:

(1) $$Y_{\mathrm{TSS}}=42.609+0.016X_{3}+0.648X_{4}-0.012X_{1}X_{4}-0.038X_{3}X_{4}-0.029X_{4}^{2}$$

**Figure 1:**
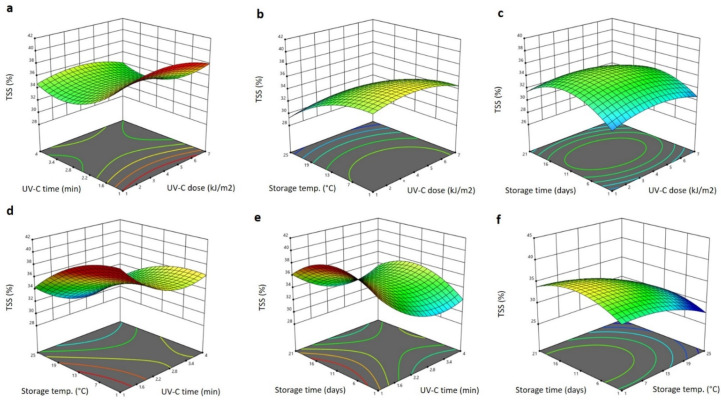
Response surface plots of total soluble solids (TSS) of Barhi dates as a function of UV-C time and UV-C dose (**a**), storage temperature and UV-C dose (**b**), storage time and UV-C dose (**c**), storage temperature and UV-C time (**d**), storage time and UV-C time (**e**), and storage time and storage temperature (**f**).

Figure 1. shows the three-dimensional (3D) response surface blot of TSS affected by the storage conditions, and the UV-C treatment and storage conditions. Increasing the UV-C dose slightly increased the TSS to the maximum level at 4 kJ/cm^2^ and then reduced it at a higher UV-C dose (Figure 1a–c). In addition, increasing the storage time greatly increased the TSS to the highest value at 15 days and then reduced it as the time elongated to 21 days (Figure 1c,e,f). However, increasing the storage temperature and the UV-C exposure time reduced the TSS of the Barhi to the minimum values at 2.8 min and 25 °C, respectively (Figure 1a,b,d–f). The increase in TSS in Barhi dates during storage time and UV-C treatment could be due to the loss of water and degradation of the polysaccharides, and similar observations were reported in Kiwi fruit treated with UV-C radiation and stored at different temperatures and durations [24]. In addition, the disruption of the cell wall matrix by UV-C might lead to the liberation of soluble solids and, in addition, might accelerate the ripening process and thereby increase the TSS of the Barhi fruits during storage. The reduction of the TSS during increased UV-C exposure time and storage temperature could be due to the inhibition of polysaccharide-degrading enzymes and thermal caramel formation [16]. When compared to the UV-C treatment dose and period, the storage temperature and time had a greater impact on raising the TSS, which suggests that the UV-C treatment slowed the decay of the Barhi dates. Similar to this, other findings suggest that mangoes’ TSS gradually increased but was slowed by the UV-C treatment, [25] likewise cherry tomatoes [26].

The significant terms of the model equation obtained for the moisture content (MC) were the linear terms of storage time and temperature (*p* < 0.05), the interaction terms of the UV-C dose and storage temperature, UV-C exposure time and storage temperature, UV-C exposure time and storage time, the storage time and temperature (*p* < 0.01) and the quadratic terms of the storage time (Table 1). Generally, the storage time and temperature reduced the MC of the Barhi in linear terms whereas, in interaction terms, different effects were observed. The polynomial equation of significant effects for the MC is:

(2) $$Y_{\mathrm{MC}}=66.095-0.415X_{3}-0.091X_{4}+0.032X_{1}X_{3}-0.049X_{2}X_{3}+0.010X_{2}X_{4}+0.087X_{3}X_{4}-0.029X_{4}^{2}$$

**Figure 2:**
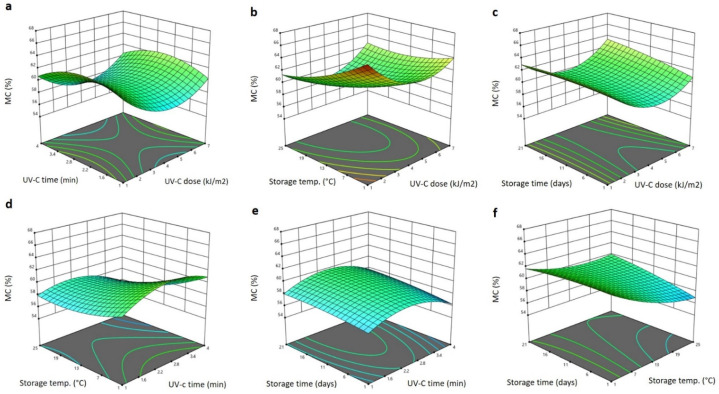
Response surface plots of moisture content (MC) of Barhi dates as a function of UV-C time and UV-C dose (**a**), storage temperature and UV-C dose (**b**), storage time and UV-C dose (**c**), storage temperature and UV-C time (**d**), storage time and UV-C time (**e**), and storage time and storage temperature (**f**).

Figure 2 displays the 3D response surface blots of the MC as affected by the storage conditions and UV-C treatment. While increasing the UV-C exposure increased the MC to the greatest level at 2.4 min and then decreased it with prolonged exposure, increasing the UV-C dosage lowered the MC of the Barhi to the lowest level, at 4 kJ/m2, and then increased it at higher UV-C doses (Figure 2a–c) (Figure 2a,d,e). The MC of the Barhi dates was gradually decreased when the storage temperature was raised, reaching the lowest values at the maximum temperature (25 °C) (Figure 2b,d,f), whereas increasing the storage time concurrently increased the MC to the maximum value at 21 days. The reduction of moisture following the treatment of Barhi dates with a moderate dose of UV-C and a high storage temperature could be due to the water loss from the surface of the date fruits by accelerating the evaporation process [27]. The increase in the MC at a prolonged exposure to UV-C and storage time is probably due to the stimulation of the lignifying enzymes, as reported earlier, that UV-C treatment increased the moisture content of peaches and apples [28]. 

### 2.3. Effect of Storage Conditions and UV-C Treatment on the Physical Characteristics of Barhi Dates

The significant terms of the RSM model for physical properties such as total color changes (Δ*E*) and firmness of the Barhi dates are presented in Table 1. The significant (*p* < 0.05) terms for firmness were the linear terms of storage time and temperature, the interaction terms of the UV-C dose and storage time, the storage temperature and UV-C exposure time, the storage time and temperature and the quadratic term of storage time. The polynomial equation for the significant terms for firmness is:

(3) $$Y_{\mathrm{Firmness}}=58.326+0.153X_{3}-1.093X_{4}+0.040X_{1}X_{4}-0.019X_{2}X_{3}-0.033X_{3}X_{4}+0.054X_{4}^{2}$$

**Figure 3:**
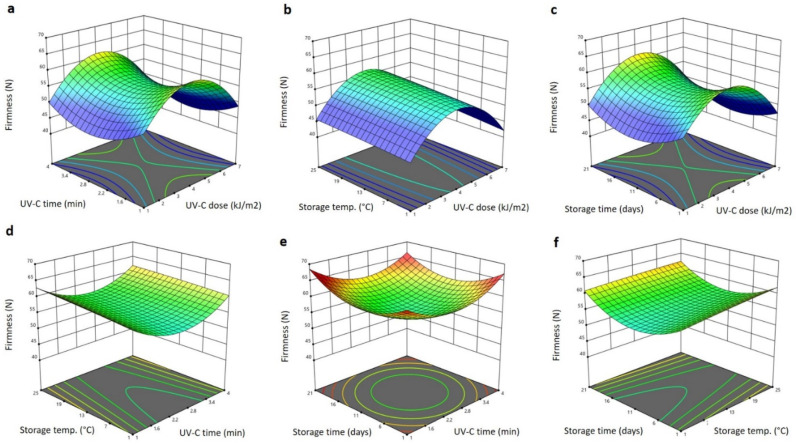
Response surface plots of firmness of Barhi dates as a function of UV-C time and UV-C dose (**a**), storage temperature and UV-C dose (**b**), storage time and UV-C dose (**c**), storage temperature and UV-C time (**d**), storage time and UV-C time (**e**), and storage time and storage temperature (**f**).

Figure 3 shows the three-dimensional (3D) response surface blot of firmness as affected by the storage conditions and UV-C treatment. Increasing the UV-C dose increased the firmness of the Barhi to the maximum levels at 4 kJ/m^2^ and then reduced them as the UV-C dose elevated to the maximum value (7 kJ/m^2^), whereas increasing the UV-C exposure time reduced the firmness to the minimum values at 2.4 min and then elevated them as the time increased (Figure 3a–e). Similarly, previous reports indicate that the treatment of various fruits and vegetables with UV-C at a dose of 2.0–4.2 kJ/m^2^ maintained the firmness during storage by eliminating the activity of cell wall-degrading enzymes [19]. Increasing storage temperature gradually increased the firmness of the Barhi, while elongating storage time initially reduced the firmness to the lowest level after 11 days of storage and then increased it as the storage duration increased (Figure 3b–f). The enhancement of the Barhi dates’ firmness with the UV-C dose and temperature increment is likely due to the adverse effect of the ultraviolet dose and heat on the cell wall-degrading enzymes, leading to a reduction in the cell wall-degradation process [25]. However, a further increase in the UV-C dose and temperature, in addition to a longer UV-C exposure time and storage duration, could lead to the physical disruption of the cell wall matrix, permitting the penetration of spoilage and fermenting microorganisms and, hence, reducing the firmness of the Barhi dates [16]. In addition, the stimulation of peroxidases, pectin methylesterase and polygalacturonases that are involved in lignification and cell wall-degradation pathways could be a cause of the reduction of firmness under the high dose UV treatment and storage conditions of Barhi dates [29].

The significant terms of the RSM model found for Δ*E* of Barhi dates, influenced by the UV-C treatment and storage conditions, were the linear terms of the storage temperature, UV-C dose and storage time (*p* < 0.05), the interaction terms of the UV-C dose and storage time (*p* < 0.01), the storage temperature and storage time and the quadratic (*p* < 0.05) terms of the storage temperature and storage time (Table 1). All other factors were insignificant and were removed from the following polynomial equation for Δ*E*:

(4) $$Y_{\Delta E}=44.695+0.385X_{1}-0.419X_{3}+0.070X_{4}+0.093X_{1}X_{4}+0.032X_{3}X_{4}+0.016X_{3}^{2}+0.043X_{4}^{2}$$

**Figure 4:**
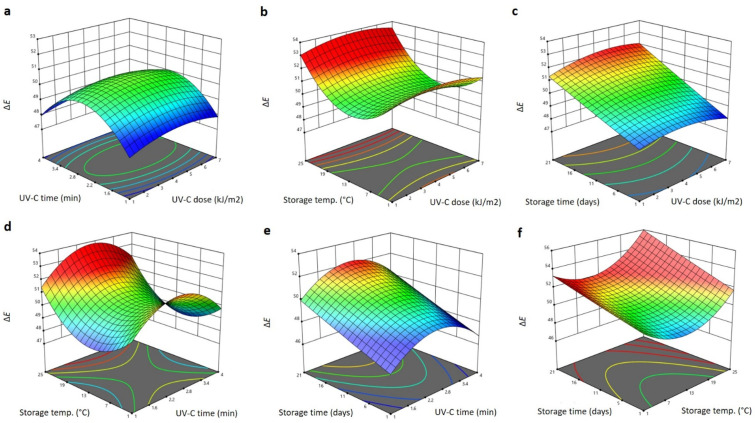
Response surface plots of total color changes (Δ*E*) of Barhi dates as a function of UV-C time and UV-C dose (**a**), storage temperature and UV-C dose (**b**), storage time and UV-C dose (**c**), storage temperature and UV-C time (**d**), storage time and UV-C time (**e**), and storage time and storage temperature (**f**).

Figure 4 depicts the 3D response surface blots of Δ*E* as influenced by the UV-C treatment and storage conditions. Increasing the UV-C dose gradually increased the Δ*E* of the Barhi dates to a high level at 4 kJ/m^2^ that was marginally reduced at higher UV-C doses (Figure 4a–c), whereas increasing the UV-C exposure time increased the Δ*E* sharply, to its highest changes at 2.4 min, and then reduced it as the UV-C treatment time elongated (Figure 4a,d,e). Increased storage time gradually decreased the Δ*E* of the Barhi dates to the maximum changes at day 21 of storage (Figure 4c,e,f), while increased storage temperature gradually decreased the Δ*E* to the lowest values at 12 °C (Figure 4b,d,f). The stimulation effects of these treatments on the chemical and enzymatic browning processes, as well as on the metabolic pathways of the phenolic compounds, oxidation and decomposition of sensitive pigments, may be the cause of the increase in the color changes of Barhi dates after UV-C treatment and storage [16,30]. Similar effects of UV-C treatments and storage on the color changes of fruits and vegetables have also been reported [16,26,30,31,32].

### 2.4. Effect of Storage Conditions and UV-C Treatment on Microbial Load of Barhi Dates

The significant terms of the RSM model recorded for the total viable count (TVC) of the Barhi as a result of the storage conditions and UV-C treatment were the linear (*p* < 0.05) terms of all factors (UV-C dose, UV-C time, storage time and storage temperature), and the quadratic term of the storage time (*p* < 0.01). Increasing the UV-C dose and storage temperature reduced the TVC, while increasing UV-C time and storage time increased the TVC, as negative and positive significant results were seen (Table 1). All other variables were not significant and were not included in the following polynomial equation for TVC:

(5) $$Y_{\mathrm{TVC}}=0.375-0.688X_{1}+1.353X_{2}-0.057X_{3}+0.079X_{4}+0.044X_{3}X_{4}$$

**Figure 5:**
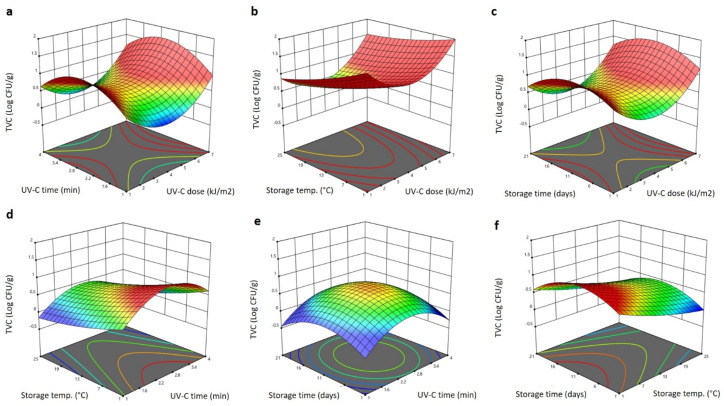
Response surface plots of total viable count (TVC) of Barhi dates as a function of UV-C time and UV-C dose (**a**), storage temperature and UV-C dose (**b**), storage time and UV-C dose (**c**), storage temperature and UV-C time (**d**), storage time and UV-C time (**e**), and storage time and storage temperature (**f**).

The 3D response surface blots generated using the RSM model and presenting the effect of the storage conditions and UV-C treatment on the TVC of the Barhi are depicted in Figure 5. Increasing the UV-C dose up to 4 kJ/m^2^ greatly decreases the TVC of the Barhi to the lowest levels, and further increases in the UV-C dose significantly increase the TVC to the highest levels at 7 kJ/m^2^ (Figure 5a–c). Increasing the storage time and UV-C exposure time increased the TVC to the highest levels at 2.4 min and 11 days, respectively, and further elongating these factors reduced the TVC of the Barhi dates (Figure 5a,c–f). Increasing the storage temperature progressively reduced the TVC of the Barhi to the lowest values at 25 °C (Figure 5b,d,f). Overall, the UV-C treatment and high storage temperature appeared to inhibit microbial growth, whereas long exposure and storage duration enhanced microbial growth on the Barhi. The decrease in the TVC of the Barhi dates following the UV-C treatment at 4 kJ/m^2^ is in accordance with the previous reports indicating that this UV-C dose is sufficient to reduce the microbial load in different fruits and vegetables [33,34]. Such a reduction was attributed to the contribution of the UV-C on the disruption of the cell membrane integrity of microbes, damaging nucleic acids and inducing mutation, thereby retarding microbial growth [32]. However, at a higher UV-C dose, longer exposure time and longer storage time, the microorganisms might show different responses due to self-adjustment or adaptation of the microorganisms to the applied UV-C dose; the aggregation of microbial cells can form shields and prevent direct exposure to UV-C, developing resistance to the UV-C [35].

### 2.5. Effect of Storage Conditions and UV-C Treatment on the Bioactive Properties of Barhi

The significant terms of the RSM model obtained for the total phenolic content (TPC) and DPPH antiradical activity of Barhi dates affected by the storage conditions and UV-C treatment showed some similarity, as shown in Table 1. The significant terms of the TPC were the linear (*p* < 0.05) terms of the UV-C exposure time, storage temperature (positive effect) and storage duration (negative effect), the interaction terms (positive effects) of the storage time and UV-C dose (*p* < 0.05), the storage time and storage temperature (*p* < 0.01) and the quadratic positive effect of the storage time (*p* < 0.05). The polynomial equation for TPC after excluding the non-significant terms is:

(6) $$Y_{\mathrm{TPC}}=2.956+1.031X_{2}+0.031X_{3}-0.089X_{4}+0.059X_{1}X_{4}+0.012X_{3}X_{4}+0.043X_{4}^{2}$$

**Figure 6:**
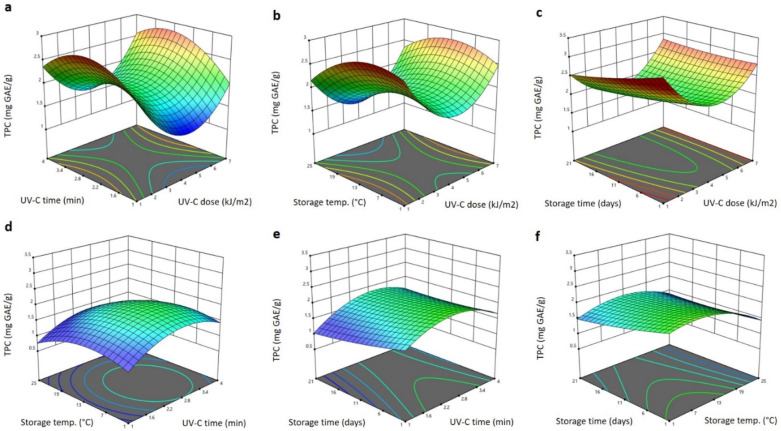
Response surface plots of total phenolic content (TPC) of Barhi dates as a function of UV-C time and UV-C dose (**a**), storage temperature and UV-C dose (**b**), storage time and UV-C dose (**c**), storage temperature and UV-C time (**d**), storage time and UV-C time (**e**), and storage time and storage temperature (**f**).

The 3D response surface blots of the TPC influenced by treating the Barhi dates with different UV-C doses for different times and subsequent storage at different temperatures and varied times are shown in Figure 6. Increasing the UV-C dose decreased the TPC of the Barhi to the minimum values at 4 kJ/m^2^ and increased it to maximum values at a high UV-C dose (7 kJ/m^2^) (Figure 6a–c). Increasing the storage temperature and UV-C exposure time increased the TPC to the maximum values at 2.4 min and 11 °C, respectively, and further elongating the UV-C exposure to 4 min and elevating the storage temperature to 25 °C resulted in a reduction of the TPC (Figure 6a,b,d–f). Elongating the storage time showed a slight reduction of the TPC of the Barhi (Figure 6). The decrease of the TPC in the Barhi dates following the treatment at 4 kJ/m^2^ is likely due to that fact that UV-C induces abiotic stress to plant tissues; to overcome such stress, phenolic compounds are exploited to counteract the stress and, consequently, their concentration declines [19]. The increase of the TPC at higher UV-C doses, UV-C exposure times and the storage temperature could be attributed to the UV-C-induction of defense mechanism (increasing the phenylalanine ammonia-lyase enzyme, de novo synthesis and accumulation of phenolic compounds), in addition to the UV-C and heat dissolution and depolymerization of the cell wall polysaccharides, thereby enhancing the release and extractability of the phenolic compounds [19,36]. In agreement with our findings, several reports indicate that the post-harvest treatment of fruits and vegetables with UV-C at different doses improved the TPC during storage at different conditions [19,26,32,33,36].

The significant terms for the DPPH antiradical activity of the Barhi dates were the linear (*p* < 0.05) terms of the UV-C dose (negative effect), storage temperature and storage duration (positive effects), interaction terms of UV-C dose and storage duration (*p* < 0.01; positive effect), storage time and temperature (*p* < 0.05; negative effect) and quadratic (*p* < 0.05) negative effect of storage duration (Table 1). All other factors were not significant and were excluded from the following polynomial equation for the DPPH antiradical activity:

(7) $$Y_{\mathrm{DPPH}}=50.761-25.763X_{1}+1.933X_{3}-0.970X_{4}+0.082X_{1}X_{4}-0.053X_{3}X_{4}-0.074X_{4}^{2}$$

**Figure 7:**
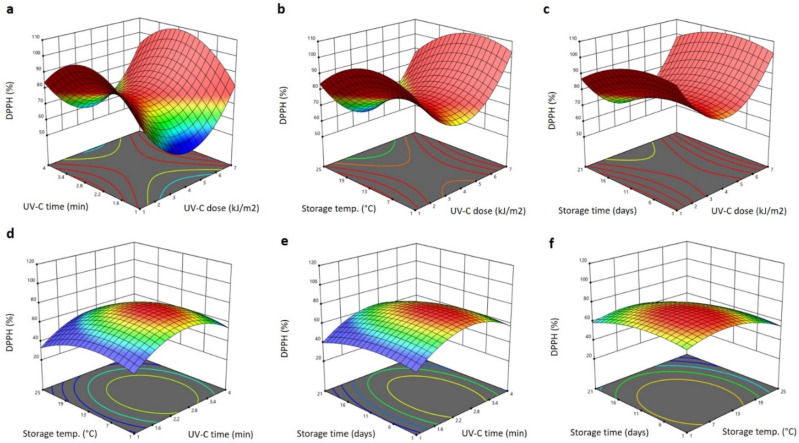
Response surface plots of DPPH antiradical activity of Barhi dates as a function of UV-C time and UV-C dose (**a**), storage temperature and UV-C dose (**b**), storage time and UV-C dose (**c**), storage temperature and UV-C time (**d**), storage time and UV-C time (**e**), and storage time and storage temperature (**f**).

The 3D response surface blots of the DPPH antiradical activity of the Barhi dates influenced by UV-C treatment followed by storage are shown in Figure 6. Increasing the UV-C dose greatly reduced the DPPH antiradical activity of the Barhi to the minimum values at 4 kJ/m^2^, which were then increased to the maximum values when the UV-C dose rose to 7 kJ/m^2^ (Figure 7a–c). Increasing the storage time, UV-C exposure time and storage temperature increased the DPPH antiradical activity to the highest levels at 2.8 min, 10 °C, and 9 days, respectively, and further increases in these factors reduced the DPPH antiradical activity of the Barhi dates (Figure 7a–f). The reduction of the DPPH antiradical activity in the Barhi dates at 4kJ/m^2^ could be due to the utilization of phenolic compounds to overcome stress induced by the UV-C treatment. Increase in the DPPH at a high dose over a long time might be due to de novo synthesis, breakdown of the cell wall and release of the bound antioxidant compounds, or the formation of antioxidant compounds, such as melanoidins [36,37]. Similar modifications in antioxidant activity following UV-C treatment and storage have previously been reported for various fruits and vegetables [32,36,37,38].

### 2.6. Effect of Storage Conditions and UV-C Treatment on the Reducing Sugars of Barhi Dates

The significant terms of the RSM model obtained for the glucose and fructose content of the Barhi dates affected by storage conditions and UV-C treatment are shown in Table 1. The significant terms for glucose were the linear terms of storage temperature (*p* < 0.05; negative effect) and storage time (*p* < 0.01; positive effect), and the interaction (*p* < 0.05) terms of the UV-C dose and storage temperature (positive effect), the storage time and UV-C exposure time (negative effect) and the storage time and storage temperature (positive effect). The non-significant terms were removed and the polynomial equation for glucose is:

(8) $$Y_{\mathrm{Glucose}}=3.594-0.375X_{3}+1.238X_{4}+0.071X_{1}X_{3}-0.111X_{2}X_{4}+0.011X_{3}X_{4}$$

The significant terms for fructose affected by the UV and storage treatments are shown in Table 1. They were linear (*p* < 0.05) terms of the storage temperature (negative effect), the interaction terms of the UV-C dose and storage temperature (*p* < 0.05; negative effect), the storage time and UV-C dose (*p* < 0.01; positive effect), the storage time and temperature (*p* < 0.05; positive effect), the quadratic terms of the UV-C dose (*p* < 0.05; positive effect) and the UV-C exposure time (*p* < 0.01; negative effect). The other factors were not significant; therefore, they were excluded from the following polynomial equation for fructose:

(9) $$Y_{\mathrm{Fructose}}=2.594-0.378X_{3}-0.054X_{1}X_{3}+0.032X_{1}X_{4}+0.085X_{3}X_{4}+1.331X_{1}^{2}-6.061X_{2}^{2}$$

**Figure 8:**
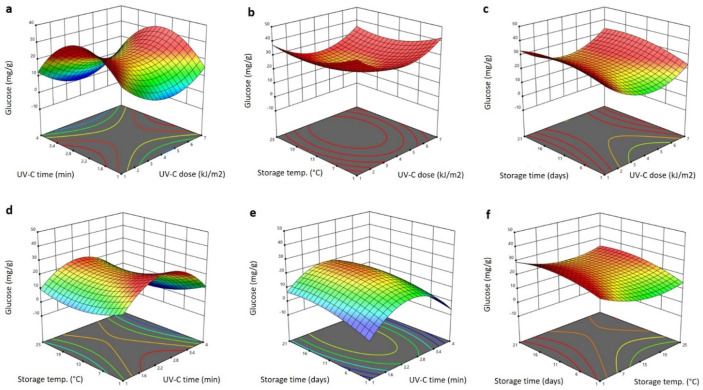
Response surface plots of glucose content of Barhi dates as a function of UV-C time and UV-C dose (**a**), storage temperature and UV-C dose (**b**), storage time and UV-C dose (**c**), storage temperature and UV-C time (**d**), storage time and UV-C time (**e**), and storage time and storage temperature (**f**).

**Figure 9:**
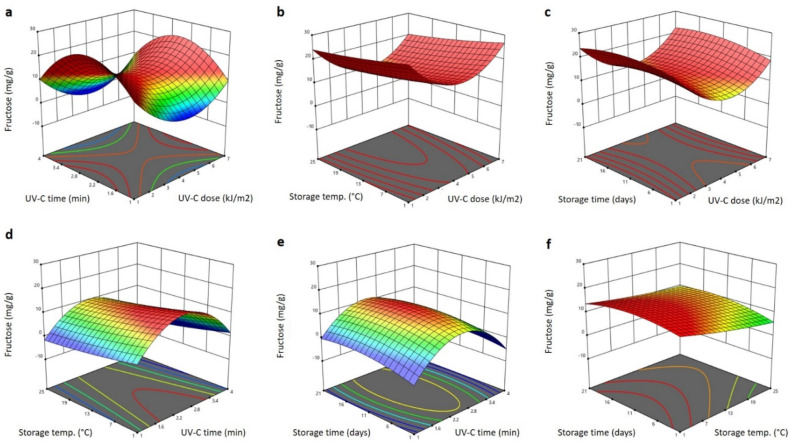
Response surface plots of fructose content of Barhi dates as a function of UV-C time and UV-C dose (**a**), storage temperature and UV-C dose (**b**), storage time and UV-C dose (**c**), storage temperature and UV-C time (**d**), storage time and UV-C time (**e**), and storage time and storage temperature (**f**).

Figure 8 and Figure 9 show the 3D response surface blots of the glucose and fructose content of Barhi dates as affected by the storage conditions and UV-C treatment. Increasing the UV-C dose reduced the glucose and fructose content to the lowest value at 4 kJ/m^2^, and then increased it as the UV-C dose was elevated to 7 kJ/m^2^ (Figure 8a–c and Figure 9a–c). On the other hand, elongating the UV-C treatment time resulted in an initial increase of the glucose and fructose to the maximum value at 2.6 min and then reduced it as the UV-C time progressed to 4 min (Figure 8a,d,e and Figure 9a,d,e). The effects of storage temperature and time on the glucose and fructose content were small, in which, the increase in storage temperature caused a slight reduction of glucose and fructose content, whereas the increase in storage time exhibited a slight increase in the glucose and fructose content of the Barhi dates (Figure 8b–f and Figure 9b–f). In agreement with our findings, several studies have shown that UV-C induces a reduction and/or increases changes in the glucose and fructose content of fruits [39,40,41,42,43]. The varied effect of the UV-C and storage on the glucose and fructose might be due to the differences in the UV type, dose, exposure time, and the fruit type and chemical composition.

### 2.7. Optimum Conditions

Based on the optimization process using the RSM model discussed in the aforementioned sections, the optimum conditions of the UV-C for preserving the quality features of the Barhi during storage were a UV-C dose of 2.07 kJ/m^2^, UV-C exposure time of 1 min, storage temperature of 12.36 °C and storage time of 18 days. The high desirability value (*D* = 1.0) demonstrated a high correlation between the predicted values and the experimental values at the optimum conditions, which were 38.24% TSS, 59.66% moisture content, 60.24 N firmness value, 48.83 Δ*E*, 0.07 log CFU/g TVC, 5.29 mg GAE/g TPC, 56.32% DPPH antiradical activity, 14.02 g/100 g glucose and 6.87 g/100 g fructose.

## 3. Materials and Methods

### 3.1. Materials

Barhi dates at the Khalal stage of maturity were purchased from selected date palm farms in the Al-Qasim and Ha’il regions in the north of the Kingdom of Saudi Arabia during the harvesting season of 2021. Cold storage trucks were used for transporting the dates from the farms to the College of Food and Agriculture Sciences, King Saud University, Riyadh on the first day of harvest. The sorting of the fruits with similar physical characteristics, followed by cleaning with compressed air, was performed upon arrival at the Laboratory. The cleaned and sound Barhi date fruits were chosen for the experiments with ultraviolet treatment and subsequent storage processes.

### 3.2. Ultraviolet (UV-C) Treatment

A UV-C system composed of a metal box with dimensions of 65 × 56 × 50 cm, a G25T8 25W T8 UV Germicidal Lamp Light Bulb 18 TUV and a lamp-holder was constructed. The UV-C lamps were attached to a flexible and resizable lamp-holder that was carefully adjusted to achieve the UV-C dose levels of 1, 3, 5, and 7 kJ/m^2^ on the surface of the fruit samples. A General Tools UV513AB Digital Meter (UVA/UVB, 280–400 nm) was used for the continuous measurement of the UV-C dose during the entire treatment process. For the treatment, a total of 48 kg of Barhi date samples was divided into 3 kg portions and then each portion was subjected to UV-C treatment at different exposure times (1, 2, 3, and 4 min) and different doses (1, 3, 5, and 7 kJ/m^2^). For the subsequent storage, the treated samples were divided into 0.3 kg portions, which were then placed in perforated plastic boxes and kept for 21 days in storage rooms set at temperatures of 1, 5, 15, and 25 °C; samples were taken at 5-day intervals for analysis of the physicochemical, microbial and nutritional quality traits.

### 3.3. Experimental Design

The optimization experiments were designed with a central composite rotatable design (CCRD) and response surface methodology using the Design Expert software version 11.0 (Stat-Ease Inc., Minneapolis, MN, USA). A four-factor [UV-C dose (X1), UV-C exposure time (X2), storage temperature (X3) and storage time (X4)] and mixed-level [X1 (1, 3, 5, and 7 kJ/m^2^), X2 (1, 2, 3, and 4 min), X3 (1, 5, 15, and 25 °C) and X4 (1, 6, 11, 16, and 21 days)] was used in the CCRD (Table 2). Six replicates at the central point were used in a total of 30 experimental runs. The moisture content, total soluble solids (TSS), total color changes (∆E), firmness, total phenolic content (TPC), DPPH antiradical activity, total viable counts (TVC), glucose and fructose were the dependent responses in the design. The independent factors in the design were the UV-C dose, UV-C exposure time, storage time and storage temperature. The second-order polynomial equation shown below was used to describe the dependent responses as a function of the independent components:

(10) $$Y=\sum \beta_{0}+\sum \beta_{i}X_{i}+\sum \beta_{\mathrm{ii}}X_{i}^{2}+\sum \sum \beta_{\mathrm{ij}}X_{i}{\;X}_{j}$$

In the equation above, the coded independent variables are X_i_ and X_j_, while the expected response, intercept and regression coefficients of the linear, quadratic and interaction components are designed as Y, β_0_, β_i_, β_jj_ and β_ij_, respectively. The F test was used to evaluate the coefficients, and surface plotting, regression and ANOVA were performed to determine the best conditions for the UV treatment and storage of the Barhi dates.

### 3.4. Mositure Content and Total Soluble Solids (TSS) Determination

The oven-drying method [44] at 105 °C was applied for the determination of moisture content of the Barhi date samples. The TSS was measured using an ABBA5 refractometer (BS instruments, Jena, Germany), as described previously [16].

### 3.5. Determination of Firmness

The Alhamdan et al. [8] approach for determining firmness was used in conjunction with a TA-HDi textural analyzer, model HD3128 (Stable Micro Systems, Surrey, England). The Barhi dates were compressed to a depth of 5 mm at a rate of 1.5 mm/s using a cylindrical probe. The force–time deformation curves, which were used to determine the firmness, were constructed using the maximum force necessary to compress the Barhi dates.

### 3.6. Determination of Surface Color

Using a HunterLab Labscan XE colorimeter (Hunter Lab, Reston, VA, USA), the Barhi dates’ CIE surface color characteristics were analyzed, as previously mentioned [16]. The colorimeter was calibrated using white plates before the lightness (*L*), yellowness (*b*), and redness (*a*) of the Barhi date CIE color characteristics were analyzed in triplicate. The following equation was used to calculate the overall color change (Δ*E*) [45]:

(11) $$\Delta E=\sqrt{{\left(\Delta L\right)}^{2}+{\left(\Delta a\right)}^{2}+{\left(\Delta b\right)}^{2}}\;$$

### 3.7. Determination of Total Viable Count (TVC)

The microbiological quality attributes of the Barhi date samples was verified by measuring the TVC, as described in the official method [44]. In this assay, the de-stoning of the Barhi date samples (25 fruits) was manually performed and then the de-stoned flesh sample (25 g) was homogenized with 0.225 L of 0.85% sterile NaCl solution. Following that, 10-fold serial dilutions were made by combining the homogenate with a sterile NaCl solution (0.85%) in a 1:9 *v*/*v* (homogenate:saline solution) ratio until adequate dilutions were obtained. The sample homogenate (1 mL) was then pour-plated on CM0309 nutritional agar media (Oxoid, Basingstoke, Hampshire, UK) and the plates were incubated at 37 °C for 24–48 h before counting the formed colonies and expressing the TVC data as the log CFU/g sample of the duplicate samples.

### 3.8. Preparation of Water Extracts of Barhi Dates

Barhi date pulp was prepared by manual removal of the date pits, and then 2 g of pulp was added to 200 mL deionized water, which was sonicated (Branson 2800 CPX ultrasound, St. Louis, MO, USA) at a 40 °C temperature for 30 min, 40 kHz frequency and 110 W constant power. After filtration (Whatman No. 1 filter paper), the filtrate was kept at −20 °C for further use.

### 3.9. Determination of Total Phenolic Content (TPC) and DPPH Antiradical Activity

The Folin–Ciocalteu (FC) reagent and DPPH methanolic solution colorimetric techniques were employed to measure the TPC and DPPH antiradical activity of the Barhi date extract, as previously described [16]. Gallic acid was utilized to create the standard curve for the TPC analysis; the sample and gallic acid’s absorbance was measured at 751 nm, and the findings were expressed as mg gallic acid equivalent GAE/g. Then, 0.1 mL of the Barhi date extract and 0.2 mL of 0.25M DPPH in methanol were combined for the DPPH antiradical activity test, and the combination was left at room temperature and in the dark for 10 min. By substituting distilled water for the extract in the preparation of the blank, the extract’s absorbance was measured at 517 nm, and the DPPH antiradical activity was estimated using the equation below:

(12) $$\mathrm{DPPH}\;\mathrm{antiracical}\;\mathrm{activity}\left(\%\right)=\frac{A_{\mathrm{control}}-A_{\mathrm{sample}}}{A_{\mathrm{control}}}$$

### 3.10. Determination of Glucose and Fructose

Using a Supelcosil LC-NH2 column (25 cm 4.6 mm 5 m) connected to a Shimadzu high performance liquid chromatography system, the glucose and fructose content of the Barhi dates was evaluated, using the previously described technique, [46] with an LC10 AD HPLC system (Shimadzu Corporation, Kyoto, Japan). The Barhi date extract was first made by mixing 10 g of de-stoned date pulp with 200 mL of ddH2O, then incubating the mixture for 30 min at 50 °C and filtering it through Whatman No.1. filter paper and then, a 0.45 μm membrane filter (Millipore, Burlington, MA, USA). Afterwards, 20 μL was injected into the HPLC column at 30 °C, and the glucose and fructose peaks were eluted at a flow rate of 1 mL/min, using a mixture of acetonitrile and water (75%:25%, *v*/*v*), and detected on a RID-10A refractive index detector (Shimadzu Corporation, Kyoto, Japan) after being diluted by 25% and 75%, respectively. The detection of the glucose and fructose was obtained by comparing their retention time with that of the authentic standard of these sugars treated and eluted in the same way as the samples. The known concentrations of glucose and fructose were used to generate the standard curves linear equations, which are used to calculate the quantity of glucose and fructose in the samples.

### 3.11. Statistical Analysis

SPSS software (SPSS Inc., Chicago, IL, USA) version 18.0 was used for the analysis of the quality attributes data of the triplicate samples, whereas Design Expert software version 11.0 (Stat-Ease Inc., Minneapolis, MN, USA) was applied for the analysis of the response surface methodology (RSM) data. The linear, interaction and quadratic effects of the UV-C dose, UV-C exposure time, storage conditions (temperature and duration), responses (firmness, total soluble solids (TSS), moisture, DPPH antiradical activity, total phenolic content (TPC), total color changes (E), total viable count (TVC), glucose and fructose) were described using analysis of variance (ANOVA). The adequacy and accuracy of the RSM models were validated using the statistical results of the adequacy, precision and coefficient indicators (CV, R^2^, and adjusted R^2^), and significance was recognized at *p* < 0.05, *p* < 0.01 and *p* < 0.001 levels.

## 4. Conclusions

This study was conducted to optimize the ultraviolet post-harvest treatment for preserving the physicochemical quality properties of Barhi date fruits at the Khalal maturity stage, at which the fruits are the most consumable and perishable; thus a proper preservation method is of high significance for Barhi date producers and consumers. In this study, a central composite rotatable design (CCRD) was used for the optimization of the UV-C dose and exposure time, and the subsequent storage temperature and duration, to increase the shelf life of the Barhi dates while mainlining the physical, chemical and nutritional quality attributes of the fruits. The results revealed that the UV-C dose and exposure time affected the quality attributes in both negative and positive manners. In addition, the storage conditions (temperature and time) influenced the quality properties of the UV-C-treated Barhi dates by different magnitudes. In general, combinations of a low UV-C dose (2.07 kJ/m^2^) and short exposure time (1 min), and a moderate storage temperature (12.36 °C), are ideal for conserving the quality attributes of Barhi dates and delaying the decay and ripening process of the fruits for up to 18 days. Overall, UV-C is an effective post-harvest method for Barhi dates that, when used at a low dose and short time, can increase their shelf life and preserve their quality.

## Figures and Tables

**Table 1:** UV Regression coefficients for process variables and product responses.

Factors	TSS	MC	Firmness	ΔE	TVC	TPC	DPPH	Glucose	Fructose
*Intercept*									
β0	42.609 ***	66.095 ***	58.326 **	44.695 **	0.375 **	2.956 ***	50.761 **	3.674 *	2.594 *
*Linear*									
X1 (β1)	1.231	−3.912	8.796	0.385 *	−0.688 *	−0.971	−25.763 *	−13.263	−12.015
X2 (β2)	−8.355	4.529	−12.409	4.580	1.353 *	1.031 *	48.046	39.532	29.469
X3 (β3)	0.016 *	−0.415 *	0.153 *	−0.419 *	−0.057 *	0.031 *	1.933 *	−1.375 *	−0.378 **
X4 (β4)	0.648 *	−0.091 *	−1.093 *	0.070 *	0.079 *	−0.089 *	0.970 *	1.238 **	0.542
*Interaction*									
X1X2 (β12)	0.011	0.071	0.169	−0.088	0.013	0.029	0.293	0.384	0.222
X1X3 (β13)	−0.072	0.032 *	0.012	0.034	0.032	0.032	−0.034	0.071 *	−0.054 *
X1X4 (β14)	−0.012 *	0.022	0.040 *	0.093 **	0.023	0.059 *	0.082 **	0.050	0.032 **
X2X3 (β23)	0.036	−0.049 *	−0.019 *	0.091	−0.020	−0.022	−0.033	−0.080	−0.022
X2X4 (β24)	0.040	0.010 *	−0.062	0.056	0.012	0.085	−0.040	−0.111 *	−0.061
X3X4 (β34)	−0.038 *	0.087 **	−0.033 *	0.032 *	0.044 **	0.012 **	−0.053 *	0.011 *	0.085 *
*Quadratic*									
X1² (β11)	−0.148	0.367	−1.255	−0.060	0.082	0.098	3.195	1.406	1.331 *
X2² (β22)	1.232	−0.893	2.520	−0.927	−0.271	−0.222	−9.145	−8.120	−6.061 **
X3² (β33)	−0.012	0.068	−0.098	0.016 *	0.060	−0.026	−0.086	0.043	0.044
X4² (β44)	−0.029 *	−0.029 *	0.054 *	0.043 *	−0.046	0.056 *	−0.074 **	−0.043	−0.024
Model F-value	76.430	123.200	3.580	71.175	81.313	41.210	31.100	71.851	4.450
*p*-value	0.001	0.0001	0.009	0.004	0.014	0.001	0.004	0.016	0.035
Mean	33.510	60.970	57.380	1.870	0.490	1.920	66.890	15.630	8.360
C.V.%	2.056	1.086	4.310	5.025	0.017	2.111	3.425	2.344	1.956
Adeq. precision	42.760	216.080	6.702	5.386	7.552	8.234	7.441	6.516	7.934
R²	0.987	0.921	0.935	0.932	0.928	0.963	0.971	0.900	0.947
Adjusted R^2^	0.927	0.879	0.874	0.890	0.882	0.811	0.832	0.862	0.884
Std. Dev.	0.617	1.005	2.470	1.791	2.310	0.011	0.245	3.660	1.640
F-value (Lack of Fit)	17.980	11.530	52.980	21.518	23.481	25.871	32.780	3.890	14.340
*p*-value (Lack of Fit)	3.037	0.193	6.720	1.504	1.001	1.042	0.987	0.106	0.128

*p < 0.05, **p < 0.01, ***p < 0.001.

**Table 2:** Independent variables and their level used for central composite design.

Independent Variables	Level				
UV dose, kJ/m^2^ (X1)	1 (−1)	3 (−0.333)	5 (0.333)	7 (1)	
UV expusre time, min (X2)	1 (−1)	2 (−0.333)	3 (0.333)	4 (1)	
Storage temperature, °C (X3)	1 (−1)	5 (−0.667)	15 (0.167)	25 (1)	
Storage time, days (X4)	1 (−1)	6 (−0.5)	11 (0)	16 (0.5)	21 (1)

## Data Availability

Not applicable.
